# Progress in Surgery

**Published:** 1900-09-15

**Authors:** 


### Progress in Surgery.

### SURGERY OF THE SPINE.

(*Continued from page 384.*)

**The Reduction of the Deformity of Spinal Caries.**—A. H. Tubby[2] recently read a paper on the Results of Manual Rectification of this deformity before the Clinical Society. Out of a first series of 25 cases which had been brought before the society in 1897, 5 had died, 4 had discontinued treatment, 15 were alive and well and able to walk, and 1 was still recumbent and sickly. The death-rate from Pott's disease had been estimated to be 25 per cent. by Billroth and others, but in the cases of Tubby and Jones it was 12 per cent. Five of the patients recovered from paraplegia as the immediate result of the operation, recurrence taking place in 1 case. In 5 cases the curvature was practically obliterated, and remained so with a spinal support, and the curve was much improved in 10 cases. A further series of 74 cases was then reported, of which 29 were walking, 28 recumbent, 7 had died, and 10 had discontinued treatment; Jones and Tubby deprecated the employment of much force, their plan having been to proceed by stages with an interval of a month between each attempt. The after-treatment should continue for three years. Their conclusions were as follows: (1) That danger to life was not great; (2) that far from producing paraplegia the operation was actually a curative measure; (3) that abscess formation was, if anything, less frequent; (4) that the risk of disseminating tubercle was a negligible quantity; and (5) that there was no risk of a flail-like spine. The contra-indications were: (1) Cervical and high dorsal curves, unless associated with paraplegia; (2) ankylosed spines; (3) large and angular deformities; (4) evidences of tubercle elsewhere; (5) in children under two years of age, and in adults over 22 years; and (6) the presence of abscess, unless there was paraplegia. They advised the operation only when the spine was yielding, and in comparatively strong children, free from abscess and tubercle. The object was not to obliterate all prominence, but to give as erect a position as possible. In the discussion, J. Jackson Clarke said he had obtained results with an antero-superior support, which compared favourably with those seen in the cases shown by Tubby and Jones. He considered that 12 per cent. was a high rate of mortality.

**Spinal Fracture and Paraplegia.**—Robert Abbe has written an article on this subject.[3] He points out that in many cases, after the damage, the displacement is spontaneously corrected in part by the rebound of the body to its natural position, so that the cord does not remain compressed; but in most cases the sharp angular deformity produced by the impaction of the fractured vertebræ holds the cord pressed against the posterior laminæ. Abbe showed a radiograph illustrating for the first time the real deformity in a case of "dislocation of the neck." These cases seldom result in a post-mortem being obtained, and it has been assumed that the characteristic deformity is due to the displacement of one articular facet backward into the notch on the upper side of the vertebræ below, thus giving the peculiar cock of the head, and slight rotation of the head to the affected side. The radiograph taken from a boy of nine shows a dislocation of the atlas upon the axis of one side. The deformity has usually been supposed to be situated between the middle cervical vertebræ. Reduction occurred spontaneously during sleep the second night after the accident. There had been little pain and no paralysis. Abbe has described three cases of broken neck from diving which he had met with that year. In case 1 there was instantaneous paralysis below the neck, but it was found that there was slight voluntary motion of the limbs, and only partial loss of sensation. For several days there was increasing and incessant jactitation of all the limbs. In three weeks the patient had excellent control of all his muscles. In case 2, that of a boy of 19, there was absolute paralysis below the neck for three days. He could feel pin-pricks, however, at some points, and there was a prickling sensation in the arms. After two days he could move the tips of the fingers and draw up the left leg. In three weeks he could walk. After six months there remained slight wrist-drop of the right hand. There was no deformity. It might well have been regarded as a case of hæmorrhage only. A radiograph, however, showed a comminuted fracture of the fifth cervical vertebræ, which had been crushed into a wedge shape, but there was less displacement forwards of the upper segment than usual, explaining why the cord was contused rather than crushed. In case 3, that of a young athlete, seen two hours after the accident, there was motor paralysis below the neck, except for the fifth root group, viz., deltoid, brachiales anticus, and triceps, giving the arms the characteristic position with the elbows held out from the sides, and the hands on the pectorals. Respiration was entirely diaphragmatic. Sensation, however, was preserved over most of the body, and he could make voluntary flexion of the two middle toes of the right foot. A radiograph showed a comminuted fracture of the body of the fifth vertebra. The posterior bony wall of the spinal canal, marked by the laminæ, was flat, and the spinous processes seemed nearly normal. It appeared that the cord was under pressure, which the removal of the posterior wall would relieve. It was not until the fifth week that operation could be undertaken, and the patient had meanwhile developed signs of local meningitis and myelitis, viz., a severe girdle pain, jactitation of the lowest unaffected muscles, and loss of any muscular action below. Acute pulmonary œdema prevented earlier operation. The operation was performed under cutaneous and deep muscular anæsthesia from cocaine. The laminæ of the fifth (fractured) and sixth vertebræ were removed. The dura was inflamed and exquisitely sensitive. After the operation the temperature dropped, and muscular power in the neck and partially in the arms rapidly returned. At the end of six months faradic irritability of all muscles except the anterior tibial groups was present, so that recovery is likely. A fourth case was related by Abbe, in which he operated for paralysis of the legs of two and a half months' duration, due to a bullet wound. There was complete loss of motion, sensation, and knee jerks. The laminæ of the last dorsal and two lumbar vertebræ were removed. A month later the patient had recovered sensation, and all the muscles were restored except those supplying the left leg and ankle. He demanded amputation of the foot a year later because it dragged. Speaking generally, Abbe states that laminectomy does not often restore power of motion, but it often relieves girdle pains, muscular twitchings, and vasomotor disturbances. The method advocated is by a straight incision six inches long to one side of the spinous processes, the knife passing between the spine and the muscles directly down to the laminæ. A blunt dissector then easily separates the muscles. The tips of the spinous processes are cut with pliers, with the interspinous ligament unbroken, and these, with opposite side muscles, are easily dissected. A rongeur is then used to gnaw away the base of the spines of as many laminæ as are required. Ashley W. Mackintosh publishes a case of fracture-dislocation of the spine, interesting in many ways, but especially for its bearing upon the localisation of fracture in the segments of the cord and in the nerve roots. As a result of a fall, a previously healthy man of 32 showed depression of the eleventh dorsal spine and prominence of the twelfth and first lumbar. From the onset there was complete paraplegia and abolition of reflexes. The upper limit of total analgesia and thermo-anæsthesia was higher than that of total anæsthesia. The anæsthesia was on the right side up to the level of the umbilicus, but on the left side two and a half inches lower. The patient died in three and a half months with uræmic symptoms and cystitis. Necropsy showed fracture-dislocation of the eleventh and twelfth dorsal vertebræ, with complete division of the cord below the origin of the twelfth dorsal roots, destruction of the upper three segments of the lumbar cord, and obliteration of the eleventh intervertebral foramen on the right side by callus and degeneration of the eleventh dorsal right posterior root, but not the left. During the clinical course of the case, although the cord had been completely divided below the twelfth dorsal roots, yet the patient sometimes felt "fluttering" sensations running down his legs, and he had vague sensations about the bladder, knowing when it was distended, and feeling some pain when the urine was being drawn off, and he seemed even to have some perception of the presence of a catheter when it was moved about. Mackintosh suggests that the fluttering sensation was due to irritation of the corresponding sensory fibres in the upper part of the cord from contraction of the cicatrix in the lower part. The bladder sensations are difficult to account for. Head is referred to as having found that in over-distension of the bladder from stricture, &c., pain is diffused as high as the eleventh segment. Perhaps the cystitis, by affecting the kidneys and ureters, caused irritation of nerves, entering the cord above the place of its division. The writer discusses the reason why, in such cases as this, the upper limit of anæsthesia is below the level of the bony lesion. Two reasons for this are given. Firstly, though the cord is crushed the roots adjacent may escape, hence they provide sensation to a level lower than that of the crush by at least the intervertebral length of the root. Secondly, Sherrington has found, by experimental section of posterior roots, that the areas of the cutaneous supply of these roots overlap so much that each area is supplied by two or three sensory roots, so that if the roots are crushed where the cord is, yet these skin areas will still retain sensation. The case under consideration illustrates this. The difference in level of the anæsthesia on the two sides in this case shows well the effect of destruction of the eleventh dorsal root on one side. There are many other interesting points in the paper of Mackintosh which we have not space to consider.

**Bullet Wounds of the Spinal Cord.**—Clinton T. Dent,[5] considering this class of injury, as seen in South Africa, says that none of the cases seen in hospital seemed more hopeless. The most remarkable feature was the astonishing rapidity with which degenerative changes set in. Sloughing sores formed in a day or two over the sacrum, and in other places where there was no pressure, cystitis sets in early, and in three or four weeks the patient runs through all the changes that in civil hospitals are accomplished only in months. An intensely acute degree of hyperæsthesia is common. The area of damage may be very great, even though the wound is transverse. In some instances, where the bullets pass close to the cord, symptoms similar to those of actual lesion show themselves at once; but after a few days, perhaps, some sensation will return, and some power of movement. The same occurs when bullets pass near nerve trunks sometimes. There is good evidence that remote structures may be violently stretched or lacerated by bullets, however cleanly they may have appeared to perforate the structures.

[2] Lancet, March, 1900. [3] Med. Rec., March, 1900. [4] Brit. Med. Jour., May, 1900. [5] Brit. Med. Jour., Feb., 1900.

### GENERAL SURGERY.

(*Continued from page* 222.)

**Fractures and Dislocations (Upper Extremity).**—Pluyette[13] has collected forty cases of simultaneous fractures of the clavicles. It is mostly met with in adult males, very seldom in women and children, and is the result of forcible transverse compression of the upper part of the thorax. There is usually much displacement and over-riding of the fragments. The shoulders are depressed and projected forwards, while the scapulæ are very prominent. Dyspnœa is frequent. The extensive over-riding of the fragments, the helplessness of the patient, and the tendency to non-union indicate in many cases the application of an osseous suture to both clavicles. This treatment was adopted with success in the case described by Pluyette.

Dr. B. F. Curtis[14] reports three cases of the rare combination of fracture of the neck of the humerus with dislocation of the upper fragment treated by operation. He thinks that after proper attempts at reduction of the upper fragment or head into the glenoid cavity under anæsthesia have failed operative measures should be resorted to, unless shock, other injuries, or extensive damage to the soft parts about the shoulder justify delay. Anterior displacements require an anterior incision; subglenoid, or posterior displacements, a posterior one, preferably by Kocher's method. Resection should be resorted to only when reduction is impossible, or when extensive damage to the parts would be required. This would probably give a better result in fracture of the anatomical neck than in that of the surgical, but reduction is to be preferred in both cases. These operations must not be undertaken except under strict aseptic conditions. Passive motion should be begun in the joints as soon as the wound has healed. The same author[15] also records an instance of fracture of the shaft of the humerus about its middle, with paralysis of the musculo-spiral nerve, in a lad aged 16. The condition of wrist drop was found two months after the injury, which had been associated with fracture of the forearm. Dr. Curtis made an incision over the fractured bone, and found the nerve stretched by the fragments and bound down by cicatricial tissue. The nerve was freed with some difficulty, but five months later almost full power in the arm and hand was regained.

Dr. Taylor[16] gives a good skiagraph of fracture of the humerus of three weeks' duration, in which there was a large amount of callus and great angular deformity. He refractured the bone, and reduced the fragment under ether. The result was perfect.

D'Arcy Power[17] recently showed before the Harveian Society a case of displacement inwards of the whole lower epiphysis of the humerus in a girl aged six years. He pointed out that it was one of a very obscure class of injuries to the elbow, the exact nature of which is only now becoming known by the application of the Röntgen rays. The skiagram showed that the separation of the epiphysis had been subperiosteal, and displaced as a whole towards the inner side. The external condyle lost its cartilaginous covering, whilst the cavity of the olecranon had its long axis oblique instead of vertical. The epiphysis appeared to be fixed in its new position by ossification of the injured periosteum and by an increased width of the internal condyle which served to support it. The arm was in a condition of cubitus varus. Mr. Power proposed to chisel away the projecting angle of the diaphysis.

As Dr. A. H. Cilley[18] says, the elbow joint is one of the most important of the whole body, and nothing short of the best possible result after fracture involving the lower end of the humerus should be allowed. If under an anæsthetic the bones cannot be satisfactorily set the joint must be promptly exposed and the reason ascertained. Under proper aseptic and antiseptic precautions we can safely and with impunity open the joint and remove the cause of the trouble. In all injuries involving the joint or very near it, the callus thrown out may mechanically obstruct the proper movements. Also with the best of care ankylosis may arise, therefore care must be taken that the bones are left in such a position (viz., the elbow flexed at a little less than a right angle) that the arm will still be useful even if the joint is stiff. Secondary operation for the removal of disability is proper when the result, from existent callus or faulty ankylosis, is not satisfactory. The locality of the incision or incisions depends on the site of trouble, which must be removed with chisel or forceps, care being taken to do no more than is necessary. The capsule is accurately closed and the soft parts brought together. In a case of backward dislocation of the ulna with epiphysial fracture of the humerus in a boy aged four years, and in another of compound dislocation of both bones, Dr. C. S. Parkhill,[19] after reduction of the dislocation, treated the joint without immobilising splints, merely bandaging them from the wrist to the shoulder with absorbent cotton and a roller smoothly applied, together with a figure-of-eight bandage round the elbow. In both cases early motion was commenced, and an excellent result ensued.

H. Littlewood[20] draws attention to a deformity of the forearm and hand, unlike the characteristic deformities and anæsthetic areas associated with musculo-spiral, ulnar, and median paralysis. When the wrist is extended, then the inter-phalangeal joints of the fingers and thumb are strongly flexed, so that the tips of the fingers touch the lower part of the palm, and no reasonable amount of force appears to be capable of straightening them. But as soon as the wrist joint is flexed to a right angle, then the inter-phalangeal joints can be easily extended. It appears always to occur in children after some severe injury about the elbow joint, and generally after a fracture separation of the lower humeral epiphysis. He has once seen it after a dislocation of both bones of the forearm backwards. It is caused primarily in the muscular portions of the flexor muscles of the forearm. These muscles have been torn, possibly by the displacement of the forearm backwards with the lower end of the humerus. The torn muscle is replaced by fibrous tissue, and this contracting is responsible for the deformity. A lump can be felt in the muscles in the upper third of the forearm, and is analogous to the well-recognised condition in the sterno-mastoid after parturition, which sometimes produces wry-neck. Littlewood relates two cases of this deformity, and describes the method which he has successfully adopted to remedy it, viz., by lengthening the superficial and deep tendons through an incision four inches long in the middle line above the wrist. The tendons are divided longitudinally for one and a half inches, and then transversely outwards and inwards above and below. The two portions are then slid one upon another and sutured.

[13] Epit. Brit. Med. Jour., Oct. 1899; and Bull. et Mém. de la Soc. de Chir., No. 2, 1899. [14] Annals of Surgery, Mar., 1900, p. 291. [15] Ibid., Feb., 1900, p. 242. [16] New York Med. Jour., Mar. 17, p. 397. [17] Lancet, Feb. 10, 1900. [18] New York Graduate, Feb., 1900, p. 193. [19] Medical Times and Register, April, 1900, p. 113. [20] Lancet, Feb. 3, 1900, p. 290. [21] The Post Graduate, Feb., 1900, p. 134.

